# Inclusive and active pedagogies reduce academic outcome gaps and improve long-term performance

**DOI:** 10.1371/journal.pone.0268620

**Published:** 2022-06-15

**Authors:** Bryan M. Dewsbury, Holly J. Swanson, Serena Moseman-Valtierra, Joshua Caulkins

**Affiliations:** 1 Department of Biological Sciences, Florida International University, Miami, FL, United States of America; 2 Department of Biology, American University, Washington, DC, United States of America; 3 Department of Biological Sciences, University of Rhode Island, Kingston, RI, United States of America; 4 Center for Teaching and Learning Excellence, Prescott, AZ, United States of America; Ryerson University, CANADA

## Abstract

We assessed the impacts of the implementation of inclusive and active pedagogical approaches in an introductory biology sequence at a large, public research university in the northeast United States. We compared academic performance between these sections with other sections of the same course where didactic approaches were used over a five-year period. We also compared this five-year period (2014–2018) with the previous five years of the same courses. Additionally, we also tracked the academic performance of the students from the sections where active learning and inclusive teaching were used, as well as the more conventionally taught (lecture-based) sections in future, mandatory biology courses. We found that the inclusively taught section of the first semester of introductory biology increased the odds of students earning higher grades in that particular section. The active learning section in the second semester narrowed the ethnic performance gap when compared to similar sections, both historically and those run concurrently. Finally, students who matriculated into the inclusively taught section of biology in the first semester followed by the active learning section in the second semester of introductory biology performed better in 200-level biology courses than students who had zero semesters of either active or inclusive pedagogy in their introductory year. Our results suggest that active and inclusive pedagogies hold great promise for improving academic performance when compared to didactic approaches, however, questions remain on the most appropriate ways for capturing the impact of inclusive approaches. Implications for institutional approaches and policy are also discussed.

## Introduction

In the last two decades STEM instruction has undergone a radical transformation in higher education. A STEM classroom experience, once assumed to be didactic, unidirectional and instructor-centered, is being reinvented on many campuses to be more student-centered and aligned with contemporary understandings of how humans learn [1]. The drivers of this change came from multiple directions. One was the plethora of federal reports calling for major upgrades in the way STEM pedagogy was administered [2, 3], in part as a need to address a workforce that was increasingly requiring more and more STEM professionals. Another was the greater attention being paid to the academic performance gaps between white students and students from historically disenfranchised identities [4]. Addressing the academic gap issue required a pedagogy that transcended blindly implementing physically active strategies and was truly inclusive of students’ voices and identities.

There is a large body of evidence suggesting that both active learning and inclusive practices (collectively referred to here as ‘learning-centered pedagogies’) can effectively improve academic outcomes [5–8]. However, these approaches have not necessarily penetrated STEM classrooms to the degree that its proponents expected [9]. There are many potential reasons why this may be the case.

First, researchers and practitioners have struggled to clearly define what active or inclusive teaching is in clearly identifiable ways [10]. Active learning for example may run the gamut from a few formative questions used during an interrupted lecture [11] to a fully flipped model where no lecturing is taking place whatsoever [12,13 but see 7]. With such a sliding scale of student engagement, clearly measuring the impact of the ‘active’ component of the pedagogy becomes difficult. Inclusive teaching has been more clearly delineated in the K12 literature [e.g. 14] but is only recently being fully unpacked and operationalized in the higher education classroom [15]. Epistemological uncertainty results in practitioners having a hard time knowing and deciding what method or suite of methods are appropriate for their context [16]. This is further exacerbated by the material supporting those texts themselves being overrepresented by dominant identities [16]. Second, as Dewsbury [17] argues, the contexts of the courses where the pedagogies are being applied matter. Adoption of learning-centered pedagogical techniques without appropriate consideration of context may lead to little positive or even harmful outcomes. Third, many aspects of learning-centered pedagogies require existing practitioners to subvert their current understanding of teaching, and learn new skills, sometimes in entirely new disciplinary areas. The infrastructure required to support this type of reform and sustain it may not necessarily be available at all institutions, thus limiting what practitioners are able to adopt and sustainably support. Fourth, the critical mass of STEM classrooms in higher education is still mostly didactic lecturing [18]. Many students matriculate into college likely assuming that this passive model is standard fare for STEM instruction. Practitioners who ask their students to actively engage in the learning process during class time sometimes face resistance, especially if the students do not fully comprehend the value of that engagement [19, 20]. Where this resistance manifests into negative course evaluations, the potential impact on the practitioners’ professional review and reward structure can cause them to shy away from future attempts of these practices. The third and fourth reasons in particular necessitate a professional structure that appropriately rewards and supports the cultivation of these practices. While some progress has been made on de-emphasizing singular evaluative components (e.g. SETS; [18]) more attention needs to be paid to creating holistic support for these practices.

The complexities and situation-specific factors associated with higher education STEM pedagogy makes the measurement of classroom interventions difficult. Studies measuring one-time interventions or reporting on small temporal scales present challenges for those interested in adoption but must translate findings for entirely new contexts. In this manuscript we report the results of the implementation of learning-centered pedagogies in two introductory biology sequenced courses (taught one after the other) at a large, public research university in the northeastern United States. We use learning-centered as an encompassing term to capture approaches that break from the conventional passive teaching approach where a single lecturer imparts information unidirectionally for the majority of the classroom time. The conventional classroom is also accompanied with a small number of high stakes assessments, with few other opportunities to demonstrate content proficiency. In the active classroom (the second semester course) the instructor used a suite of evidence-based methods (including the use of small-groups, interrupted lectures and in-class problem solving) to ensure that students were full participants in their learning process. The inclusive teaching course (the first semester Biology) in this study follows the Deep Teaching framework outlined in Dewsbury [21] and Dewsbury and Brame [22], where specific pedagogical approaches and strategies are enacted as a function of carefully cultivated relationships and continuous dialogue with the students. While an inclusive classroom incorporates several active learning methods, the dialogic relationship is the key driver of the classroom experience. We were interested in how five years (2014–2018) of utilizing these approaches impacted 1) academic outcomes between ethnicities within the courses where learning-centered pedagogies were used, 2) academic outcomes compared to didactically taught sections at the same time, 3) academic outcomes compared to the previous five years of instruction (also lecture-based) of the same courses, and 4) future academic performance of students who, after the introductory biology level matriculate into upper division biology courses. We also contextualize our findings within the institution where the study took place and discuss several nuances of the praxis of inclusive and active approaches.

## Materials and methods

### Institutional context

The study was conducted at a large, public research university in the northeast United States and approved by the institution’s Institutional Review Board (#858301) from their Office of Integrity which is housed in the Office for Sponsored Research. Informed consent was waived by the IRB as this involved the gathering of archived data. Data was anonymized and coded with a numeric identifier so that the identities of the individual students were unknown to the researchers. The institution is predominantly white, with approximately 23% of the undergraduate population identifying as PEERS (Persons excluded because of ethnicity or race, [23]). Principles of Biology is a high-enrollment two-semester survey course that is offered from the Department of Biological Sciences. The first semester (Principles of Biology I) is taken by biology majors as well as several other STEM majors for whom the course serves as a prerequisite for other courses. The second semester is taken by biology majors only. Majors and those requiring the prerequisite are usually unevenly spaced between the four sections of the first semester. The first semester surveys the fundamentals of cells and molecules, molecular biology, populations genetics and anatomy and physiology. Students needing the second semester of Principles of Biology must earn a C or above in order to matriculate into that course (Principles of Biology II). Principles of Biology II focuses on evolutionary biology, plants and animal ecology. There are two sections of this second-semester course. Most students take the two-semester sequence during the Fall and then Spring semesters. The learning outcomes for both courses are generally similar between sections, but each instructor is allowed to operationalize those learning outcomes apropos to their personal pedagogical style. However, the institution also offers these courses as a Spring to Fall sequence. We focused on the Fall to Spring sequence in this study.

Prior to 2014, there were only three, simultaneously taught sections of the Principles of Biology I taught by different instructors, all of which were taught using a didactic, lecture-based style. While there were small differences between instructors’ styles, in general, all heavily relied on multi-slide PowerPoint presentations, with little to no formative assessments embedded in the pedagogy. Summative assessments happened four to five times per semester and were usually the only mechanism for students to earn credit toward the final course grade. Principles of Biology II was also mostly taught using a primarily lecture approach until the 2014/2015 academic year. In 2014, a new instructor was hired, and a new section of Principles of Biology I was offered. The new instructor (first author Dewsbury) piloted what Dewsbury [21] refers to as ‘Deep Teaching’, an inclusive approach centered around the dialogic relationship between instructor and student. In this approach, an elaborate, ongoing process is enacted to fully understand the psychosocial contexts of the students who are entering the classroom each semester the course is offered. Decisions around active-learning strategies, support structures, and all other curricula nuances are driven by how this understanding unfolds and evolves. For example, even seemingly simple decisions such as the length of videos watched in the flipped classroom model is impacted by the instructor’s knowledge of the work hours the students keep. While this approach necessitates the use of several active learning approaches, the centrality of the student voice in driving the process separates it as a fully inclusive practice. The three other sections of Principles of Biology I continued using a lecture-based approach. Of the two sections of Principles of Biology II, one transformed their section to incorporate several active learning approaches beginning in 2013. Students were placed into small groups where several activities were used to engage them in the learning process, provide multiple opportunities for formative assessment, and in general move the classroom away from passive approaches to learning. While the practices used were refined over time, we characterize this classroom as active and not inclusive largely because it did not follow the specifications pertaining to dialogue outlined in the Dewsbury [21] framework.

The truly inclusive classroom begins with recognizing how the instructor, and their social positioning play a key role in cultivating an inclusive environment. This self-awareness includes revisiting their own pathways through the profession, degree of knowledge about social structures and an understanding of their role in education beyond subject-area expertise. This step requires at the very least an understanding of the unconscious biases we all have, as shaped by our personal social histories, and recognizing the ways in which those biases may play out in classroom dynamics. The inclusive practitioner commits to self-education, particularly in areas of social structures about which they were previously unaware in order to interrogate their own ideologies, and to better understand the social contexts from which students arrive into the classroom [e.g. 24]. Cultivating this macro understanding of social context is then followed by specific approaches to get to know the students more personally. For example, the inclusive classroom uses a reflection assignment called ‘I believe’, using the exact prompt from the National Public Radio (NPR) weekly program (http://thisibelieve.org). These reflection essays provide a window into the soul of the students, and after sharing their own essay, the dialogic relationship between instructor and student begins. Reflection essays also provide key clues into students’ mindset, economic and identity contingencies and general beliefs about their personal strengths. This is informative, since it lets the instructor know that instructional practices that promote growth mindsets, confidence and increased task value, are crucial before content specific strategies are enacted. Another specific approach enacted in response to a better understanding of the students is the course’s assessment structure. The course provides several opportunities for students to demonstrate proficiency including group assignments and pre-class assignments. More importantly the summative assessments (four) are each only worth 15% of the final grade. This is a significant enough percentage such that the performances on the individual summative assessments are mathematically consequential for the final grade. However, it is low enough that if there are initial struggles there are sufficient opportunities available for a pathway back for success in the course. This is particularly important in the early stages since early struggles may be more indicative of identity contingencies, college transition struggles or other social factors, none of which speak to the student’s actual ability to do biology. Therefore, an inclusive assessment structure does not penalize students for social factors over which they have little control and provides the instructor an opportunity to mitigate those factors and help the students find their academic feet.

In this study, we are making a historical comparison between sections that used different pedagogical approaches. As a result, we are unfortunately unable to account for any differences between instructor styles (however small) of the lecture sections and/or the way in which assessments were administered in those sections. However, tracking all students to the 200-level classes allowed for a direct comparison of their academic proficiency regardless of the history of their instruction.

### Statistical methods

For this quasi-experimental study, data was obtained from the Office for Institutional Research and included Principles of Biology I final course grades and course section number, high school grade point average (HSGPA), combined SAT scores (SAT), first generation status (FG), and ethnicity (underrepresented minorities, URM) for students enrolled during fall semesters from 2009–2018. Any subsequent biology final course grades were also collected for these students, including Principles of Biology II. During 2009–2018, SAT exams changed formats; for comparison purposes, conversions were made to the newest version’s combined score according to concordance tables found on the College Board website [25]. Analyses of variance (ANOVAs) were run in SAS, version 9.4, followed by Tukey-HSD post-hoc comparisons and used to evaluate possible performance gaps in Principles of Biology I and future course outcomes. However, due to a non-normal distribution for final course grades in Principles of Biology II, a non-parametric Kruskal-Wallis test was used followed by DSCF post-hoc comparisons of pairwise Wilcoxon ranked-sum differences. To compare academic outcomes between the treatment section of Principles of Biology I and the didactic sections, an ordinal regression model was run using SPSS, version 26, with final course grade as the dependent variable. The model included student-level descriptive predictors, URM and FG status, student-level measures of academic preparedness, HSGPA and SAT, and treatment. Correlations were determined to identify any collinearity and possible overfitting of the model. Intercorrelation coefficients (ICCs) and a vector of fixed effects were also calculated to evaluate the impact of clustering in the data. Due to large ICCs, a complex sample was designed in SPSS with clusters created for the year the data was collected and for course section. The ordinal regression was then conducted using this complex sample design.

## Results

Overall, learning-centered pedagogies appeared to have varied effects on students’ academic performance. Results reported here are informed by Wassertein and colleagues’ [26] guidance on how probability values and confidence intervals should be interpreted and described. This article (and several others in a special issue of American Statistician) addresses the overuse and misuse of the term significance and over-reliance on p-values as indicators of treatment effects. Our reporting here attempts to partially comply with their advocacy of greater data transparency and a more comprehensive presentation of our quantitative findings. Therefore, we still report statistical significance test results (p-values) *and* describe overall trends seen in our data.

### Academic performance gaps between ethnicities in learning-centered pedagogy classrooms

**Fig 1:**
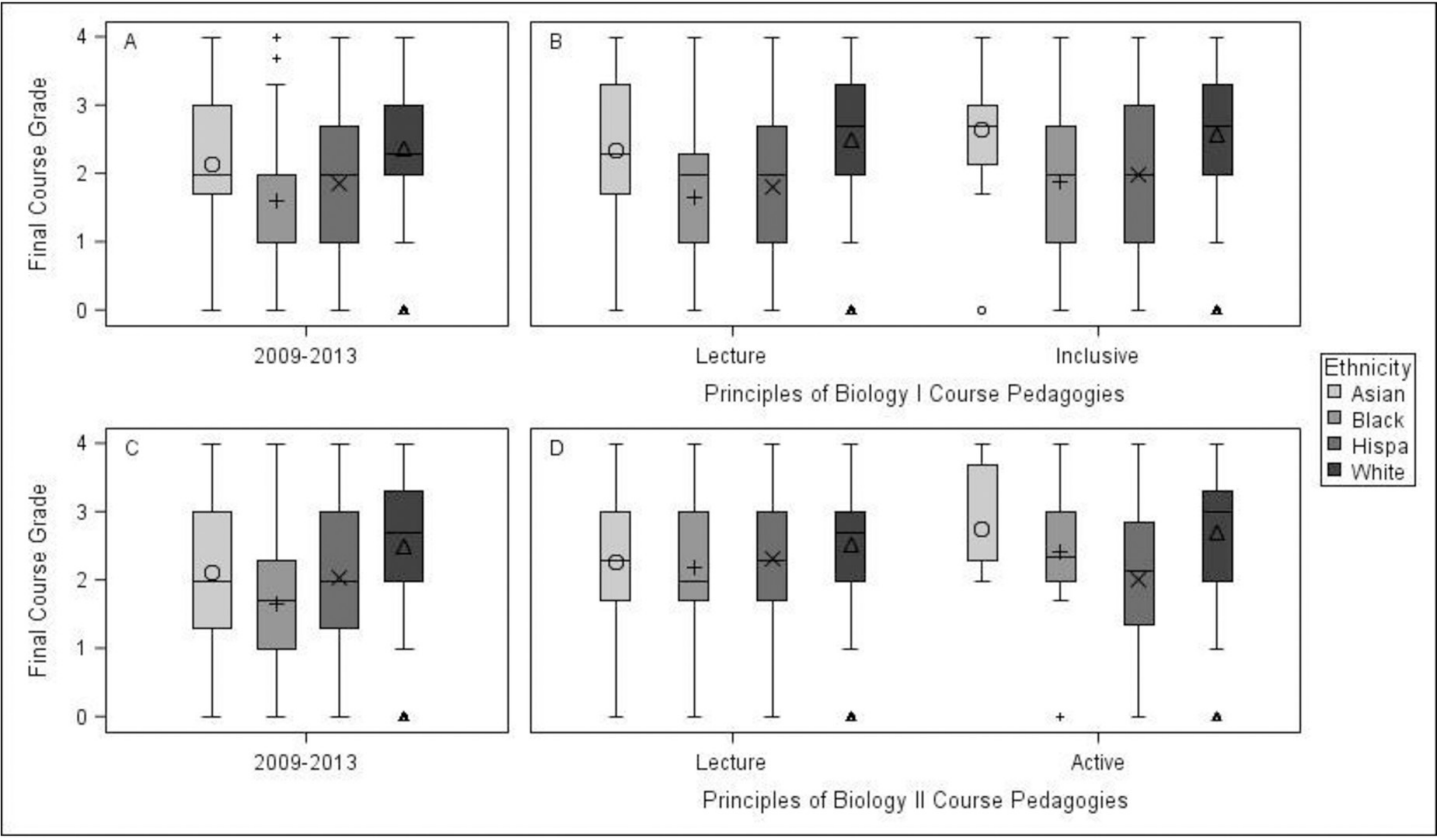
Panel A: Analysis of variance detected achievement gaps in Principles of Biology I between 2009–2013 (F(3, 4423) = 62.75, p < .0001, R^2^ = 0.041, 95%CI [0.030, 0.052]). (Demographic group sizes, n = 198 Asian, 269 Black, 421 Hispanic, and 3702 White) Panel B: ANOVA detected medium differences in final grades for the first introductory biology course during 2014–2018 between ethnicities for the lecture-styled sections (F(3, 3398) = 70.88, p < .0001, R^2^ = 0.059, 95% CI [0.044, 0.074], Cohen’s d = 0.68 (White and Black students), 0.57 (White and Hispanic students)) and the inclusive section (F(3, 565) = 10.61, p < .0001, R^2^ = 0.053, 95% CI [0.017, 0.089], Cohen’s d = 0.58 and 0.55 respectively). (Demographic group sizes for lecture sections, n = 172 Asian, 217 Black, 426 Hispanic, and 2,705 White; Demographic group sizes for inclusive treatment, n = 17 Asian, 42 Black, 70 Hispanic, and 427 White). Panel C: A Kruskal-Wallis Test detected achievement gaps in Principles of Biology II between 2009–2013 (χ^2^ (3, N = 1889) = 76.513, p < .0001, Cramer’s V = 0.116). (Demographic group sizes, n = 80 Asian, 94 Black, 188 Hispanic, and 1527 White) Panel D: In the second course in the introductory biology sequence, Kruskal-Wallis Tests detected small differences between grades as a function of student ethnicity in the lecture sections (χ^2^ (3, N = 832) = 9.924, p = 0.019, Cramer’s V = 0.063) but not in the active learning section (χ^2^ (3, N = 249) = 7.099, p = 0.069, Cramer’s V = 0.097). (Demographic group sizes for lecture sections, n = 37 Asian, 42 Black, 84 Hispanic, and 576 White; Demographic group sizes for active sections, n = 7 Asian, 10 Black, 16 Hispanic, and 216 White).

One-way ANOVAs were conducted to determine the appropriateness of combining sections for subsequent analyses at the student level. Based on academic preparedness measures, the treatment section was possibly at an academic disadvantage; SAT: F(1, 4295) = 2.66, p <1.03, and HSGPA: F(1, 4169) = 13.06, p < .0001 where the mean HSGPA for the lecture sections was 3.44 (SD = 0.50) and 3.36 (SD = 0.46) for the treatment section. Academic performance was measured by final grade earned in the course, which was converted to the GPA points that corresponded to the letter grade. In accordance with the institution’s policies, A was equal to 4.0 GPA points, A- = 3.7, B+ = 3.3, B = 3.0, B- = 2.7, C+ = 2.3, C = 2.0, C- = 1.7, D = 1.0 and F = 0 points. Over the 2014–2018 five-year period, White and Asian students earned the highest median GPA points in the inclusively taught section of Principles of Biology I (F(3, 565) = 10.61, p < .0001; Fig 1, Panel B). Both earned, on average, almost the equivalent of a B grade, though the range of grades earned by Asian students was much smaller than those of White students. There were also far fewer Asian-identified students who enrolled in this course than students from any other self-reported identity. Students identifying as Black or Hispanic in Principles of Biology I averaged a C over the five years in the section where inclusive approaches were used. The interquartile range of grades of Hispanic students were slightly larger than those of Black students, and both these groups’ interquartile ranges were much larger than those of White and Asian students. Median grade GPA values in the Principles of Biology II course where active learning was used (Fig 1, Panel D) ranged between 3.0 (White students) to 2.2 (Hispanic students) [χ^2^ (3, N = 249) = 7.099, p = 0.069]. The interquartile ranges for Black and Hispanic students were less than those of White and Asian students. Both Black and Hispanic student groups’ interquartile range included grades below the passing grade (C) for the course.

### Academic performance in learning-centered pedagogy classrooms versus lecture-based approaches

Correlations between variables were calculated to ensure a lack of collinearity and overfitting of our model. The strongest correlation was detected between course section and treatment, r = 0.655, which is to be expected based on the dummy coding of treatment based on course section. The next strongest correlations were between final course grade and SAT score (r = 0.508) and high school GPA (0.480). An intraclass correlation coefficient (ICC) was calculated of each of the models independent variables with the outcome variable, final grade. Approximately 44% of the variation observed in the data is found between sections therefore, a complex sampling protocol was created in SPSS to account for clustering of data by year and section.

Overall, the odds for students enrolled in the inclusive section to earn one grade interval higher (ie. B to a B+) are 2.287 (95% CI [1.566, 3.340]) times when compared to the lecture sections when all other variables are held constant (Table 1). There were academic performance gaps in lecture-based classrooms in Principles of Biology I between 2014 to 2018 (F(3, 3398) = 70.88, p < .0001, Fig 1, Panel B). Black students’ interquartile range of grade values on average were in the failing range during this entire period while the interquartile range of the white students was entirely within the passing range. This was the largest difference between any two ethnicities (MD = 0.870, 95%CI [0.632, 1.108]). The interquartile range was largest for Hispanic students during this period. White students scored higher than students of other ethnicities in the lecture-based section of Principles of Biology II (Χ^2^ (3, N = 832) = 9.924, p = 0.019, Fig 1, Panel D). They had higher median values and a smaller interquartile range when compared to other ethnicities. With the exception of Hispanic students, each ethnicity earned lower academic grades in this section than their counterparts where active learning approaches were used.

**Table 1:** Ordinal regression model for final grade in Principles of Biology I with point estimates, standard errors, and odds ratios (N = 2877, df = 19).

Variables	95% CI for parameter estimates	OR
URM	(-0.590, 0.143)	1.25
FG	(0.020, 0.341)	0.835*
HSGPA	(1.464, 1.916)	0.185*
SAT	(0.005, 0.007)	0.994*
Treatment	(-1.206, -0.448)	2.287*
URM* Treatment	(-0.052, 0.910)	*
FG* Treatment	(-0.243, 0.220)	
pseudo-R^2^	0.383	

*p < .05, Nagelkerke pseudo R-squared reported.

When first-generation status was removed from the analysis, sample size increased to 3927 students. When clustered by year and section, the treatment yielded a smaller odds ratio, 1.727 (95%CI [1.059, 2.818]). Additional analyses can be seen in the supplemental tables (S1 File).

### Comparison to preceding five years (2009–2013)

In Principles of Biology I, the performance of different ethnic groups followed a mostly similar pattern taught in the five years preceding 2014. White students in general earned the highest grades while Black students earned the lowest (F(3, 4423) = 62.75, p < .0001, Fig 1, Panel A).

Academic performances of students in Principles of Biology II of the 5 years preceding 2014 were very similar to the 2014–2018 period (Fig 1, Panel C). In both cases Black students averaged a failing grade for the class over the entire time period, while the other ethnic groups averaged between a C and a B during the same period. The grades of Black, Asian, and Hispanic students ranged the entirety of A to an F during both periods, but that grade range was similar for whites during the 2009–2013 period. In the active learning section of Principles of Biology II, the 2014–2018 period saw a closing of the academic performance gap between Black students and White students as well as Black students and Hispanic students that existed during the 2009–2013 period. There was also a closing of the gap between Asian students and White and Hispanic students. The only group whose average grade decreased during the 2014–2018 period was Hispanic students, but their median grade was still at a C, the passing grade for the course.

### Subsequent academic performance

**Fig 2:**
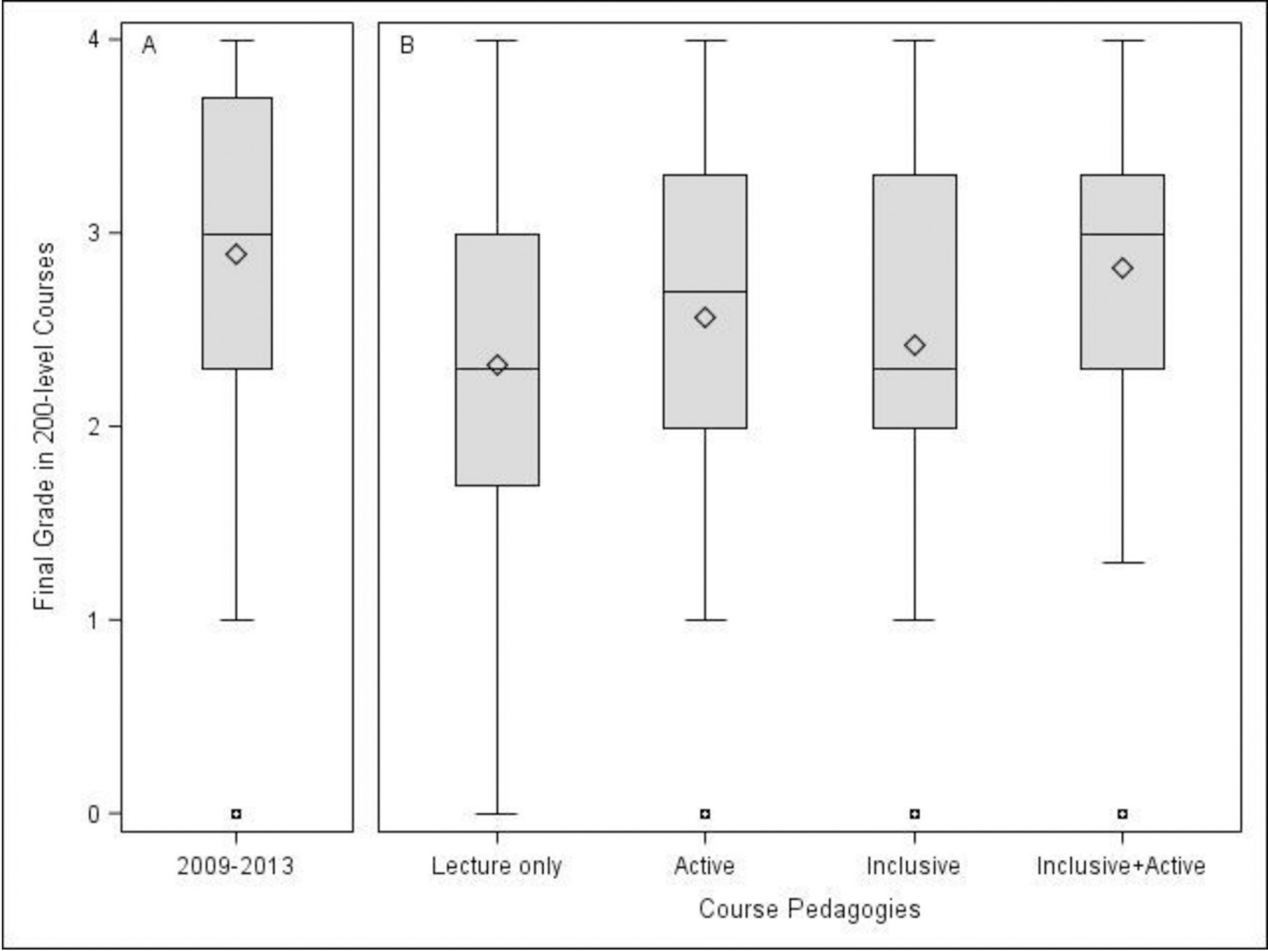
There was no difference between treatments and mean final course grades in 200-level biology courses over 2014–2018 (F(3, 239) = 2.17, p = 0.092, R^2^ = 0.027, 95% CI [-0.013, 0.067]). Although data suggests there may be a small difference between the group who took the active and inclusive section both semesters (N = 23, M = 3.030, SD = 0.657) and the group who took them neither semester (N = 128, M = 2.541, SD = 0.907), F(1, 149) = 6.12, p = 0.015, R^2^ = 0.039, 95% CI [-0.021, 0.099].

The impact of utilizing learning-centered pedagogies was also seen in how students performed in the first set of upper division courses they took after the Principles of Biology I and II series. Students who matriculated into 200-level classes from the Principles of Biology I and II series from the 2009–2013 (Fig 2, Panel A) had similar academic outcomes to students who took both the inclusive teaching section of Principles of Biology I and the active learning section of Principles of Biology II. These students also scored the highest median grades in 200-level courses compared to the other amounts of exposure to learning-centered pedagogies. This was followed by students who took lecture-based sections of Principles of Biology I followed by the active-only section of Principles of Biology II. Students who took the inclusive-only section of Principles of Biology I followed by the lecture-based section of Principles of Biology II scored third lowest, and students who had no exposure to neither learning-centered pedagogy earned the lowest grades.

## Conclusions

Our exploration of five years of learning-centered pedagogies in an introductory biology course sequence yielded several lessons pertaining to how we consider general performance, as well as disaggregated performance in introductory STEM courses. Overall, we submit that the findings from the analysis of quantitative academic outcomes posits more questions than they answer. We lay these out below, in the context of existing literature pertaining to the social contexts of learning.

### Academic performance and outcome gaps in introductory STEM courses

Our data suggest that the academic performance of students in the inclusively taught section of Principles of Biology I resulted in better performance of students in this section overall as they were more likely to get an A or B and least likely to earn a DFW grade. With the exception of black students in the lecture sections during the 2014–2018 period, outcomes patterns were overall similar to those in the time period before this pedagogy was introduced. In those sections Black students averaged a failing grade. This suggests that the key difference driving the improved overall performance was the improved outcomes for Black students. In the second semester of this introductory course however, the active learning section reduced the major differences in the performance of Black and Asian students when compared to both previous iterations of this section, *and* the lecture-based section taught during the same semester.

The implementation of the inclusive approach was in response to the nagging academic performance gaps between different ethnic groups. The persistence of these patterns potentially speaks to the possibility that there are non-cognitive factors impacting the academic experience for different individuals and groups in different ways. Quantitative explorations such as this study sometimes implicitly assume that regardless of the student’s starting point, in-class instruction will singly drive all students to academic proficiency. The literature on the social context of education suggests that we should reconsider that argument. It is well-documented that metrics such as SAT score and HSGPA correlate strongly with socioeconomic indicators of privilege [27–29]. Additionally, the transition to college for many students, especially those from historically disenfranchised identities may be exceptionally difficult due to issues pertaining to social belonging and reduced social capital [30]. If SAT and HSGPA predict success in an introductory biology course, then, it is unclear if that prediction is an indication of actual preparedness or that of an unknown non-cognitive social factor. Our data bears this out somewhat. If inclusive approaches (Principles of Biology I, section 4) reduce the predictive power of these variables, particularly for Black students, they may enact the very thing inclusive spaces are meant for–making negative socioeconomic indicators less relevant. While the impacts of this were reflected in content gains for black students’ academic outcomes data do not fully capture the impact in terms of sense of belonging and affirmed identities. The relationship between inclusive approaches and the cultivation of these constructs should be an immediate area of research exploration.

In a similar vein, the narrowing of the achievement gap in the second semester of the introductory biology sequence is potentially a reflection of a more equal academic starting point for students successfully entering the course. One can hypothesize that students acclimated to the learning-centered pedagogical class experience at that point are less likely to be impacted by the negative externalities associated with the college transition process.

Overall, our data indicates that students benefit from the combination of the two courses taught using learning-centered pedagogies. This is important since STEM instruction is still largely didactic, and, as discussed previously, some hesitation still exists for the adoption of these methods. In this light, two concerns typically raised by practitioners include 1) the potential negative effects of content reduction to allow time for more activities [31] and 2) the perception that inclusive approaches are considered ‘over coddling’ of students [32]. We do not have space in this manuscript to fully address these concerns, but the fact that students from learning-centered pedagogy classrooms perform equally well or better in upper division courses should allay fears of negative impacts associated with content reduction.

### How the academic experience is measured

Though our findings indicate that Deep Teaching can engender success in upper division classes, new questions, separate from the primarily academic measures discussed in this manuscript should be considered. Some reflection is necessary on what constitutes success in an educational experience, the components of that experience, and how the process is assessed. More specifically, inclusive teaching as discussed in this manuscript is built on the Freirean concept of dialogue [33], and the more recent theory of emerging adulthood [34]. At their core, both speak to inclusively minded practitioners facilitating meaning-making as a key component of the developmentally appropriate process of identity exploration. Some studies have shown a link between the affirmation of STEM identity and academic performance [32], but in this context, we are referring to meaning-making as an end unto itself. If current pedagogical assessment and research questions solely focus on content gains, how then is this meaning-making, a critical component of inclusion, captured as an effect of inclusive practice? Our point here is not to be dismissive of the importance of content mastery. But as we shift our paradigms to considering inclusive approaches as an expectation, the ways in which we assess the academic experience likely must expand or change.

As a crucial entry point into the STEM pathway, introductory biology can serve as an encouraging open door or an unwelcome sieve. Our exploration of 5 years of implementing learning-centered pedagogies meant to welcome suggests that much success can be gained from these approaches. However, our work also underscores that there is still much unknown about engendering success for students in the first critical semesters, and that new and more diverse metrics are needed to fully capture the impact of inclusive practices.

## Supporting information

S1 Dataset (XLSX)

S1 File (DOCX)

## References

[1] Borrego Maura, and Henderson Charles. "Increasing the use of evidence‐based teaching in STEM higher education: A comparison of eight change strategies." *Journal of Engineering Education* 103, no. 2 (2014): 220–252.

[2] American Association for the Advancement of Science. "Vision and change in undergraduate biology education: A call to action." *Washington*, *DC* (2011).

[3] National Research Council. *BIO2010*: *Transforming undergraduate education for future research biologists*. National Academies Press, 2003. 20669482

[4] Gonzalez Heather B., and Jeffrey J. Kuenzi. "Science, technology, engineering, and mathematics (STEM) education: A primer." Washington, DC: Congressional Research Service, Library of Congress, 2012.

[5] Freeman Scott, et al. "Active learning increases student performance in science, engineering, and mathematics." *Proceedings of the National Academy of Sciences* 111.23 (2014): 8410–8415. 10.1073/pnas.1319030111 PMC4060654 24821756

[6] Schmid Megan E., et al. "Promoting student academic achievement through faculty development about inclusive teaching." *Change*: *The Magazine of Higher Learning* 48.5 (2016): 16–25.

[7] Theobald E.J., Hill M.J., Tran E., Agrawal S., Arroyo E.N., Behling S., et al., 2020. Active learning narrows achievement gaps for underrepresented students in undergraduate science, technology, engineering, and math. Proceedings of the National Academy of Sciences, 117(12), pp.6476–6483. 10.1073/pnas.1916903117 PMC7104254 32152114

[8] Ballen C.J., Wieman C., Salehi S., Searle J.B. and Zamudio K.R., 2017. Enhancing diversity in undergraduate science: Self-efficacy drives performance gains with active learning. CBE—Life Sciences Education, 16(4), p56. doi: 10.1187/cbe.16-12-0344 29054921 PMC5749958

[9] Brownell Sara E., and Kimberly D. Tanner. "Barriers to faculty pedagogical change: Lack of training, time, incentives, and… tensions with professional identity?." *CBE—Life Sciences Education* 11.4 (2012): 339–346. doi: 10.1187/cbe.12-09-0163 23222828 PMC3516788

[10] Keyser Marcia W. "Active learning and cooperative learning: understanding the difference and using both styles effectively." *Research strategies* 17.1 (2000): 35–44.

[11] Jenkins Alan. "Active learning in structured lectures." *Teaching large classes in higher education*: *How to maintain quality with reduced resources* (1992): 63–77.

[12] Bishop Jacob Lowell, and Matthew A. Verleger. "The flipped classroom: A survey of the research." *ASEE national conference proceedings*, *Atlanta*, *GA*. Vol. 30. No. 9. 2013.

[13] Moog Richard S., James N. Spencer, and Andrei R. Straumanis. "Process-oriented guided inquiry learning: POGIL and the POGIL project." *Metropolitan Universities* 17.4 (2006): 41–52.

[14] Timken Gay L., and Watson D. "Teaching all kids: Valuing students through culturally responsive and inclusive practice." *Standard-based physical education curriculum development* 2 (2010): 122–153.

[15] Driessen Emily P., Jennifer K. Knight, Michelle K. Smith, and Cissy J. Ballen. "Demystifying the meaning of active learning in postsecondary biology education." CBE—Life Sciences Education 19, no. 4 (2020): ar52. doi: 10.1187/cbe.20-04-0068 33001767 PMC8693947

[16] Simpson Dasia Y., Abby E. Beatty, and Cissy J. Ballen. "Teaching between the lines: Representation in science textbooks." Trends in Ecology & Evolution 36, no. 1 (2021): 4–8. doi: 10.1016/j.tree.2020.10.010 33187728

[17] Dewsbury Bryan M. "Context determines strategies for ‘activating’ the inclusive classroom." *Journal of microbiology & biology education* 18.3 (2017). doi: 10.1128/jmbe.v18i3.1347 29854055 PMC5976051

[18] Hadad Y., Keren B. and Naveh G., 2020. The relative importance of teaching evaluation criteria from the points of view of students and faculty. Assessment & Evaluation in Higher Education, 45(3), pp.447–459.

[19] Stains Marilyne, et al. "Anatomy of STEM teaching in North American universities." Science 359.6383 (2018): 1468–1470. doi: 10.1126/science.aap8892 29599232 PMC6310123

[20] Deslauriers Louis, Logan S. McCarty, Kelly Miller, Kristina Callaghan, and Greg Kestin. "Measuring actual learning versus feeling of learning in response to being actively engaged in the classroom." Proceedings of the National Academy of Sciences 116, no. 39 (2019): 19251–19257. doi: 10.1073/pnas.1821936116 31484770 PMC6765278

[21] Dewsbury Bryan M. "Deep teaching in a college STEM classroom." Cultural Studies of Science Education (2019): 1–23.

[22] Dewsbury Bryan, and Cynthia J. Brame. "Inclusive Teaching." CBE—Life Sciences Education 18.2 (2019): fe2. doi: 10.1187/cbe.19-01-0021 31025917 PMC7058128

[23] Asai David. "Excluded." Journal of Microbiology & Biology Education 21, no. 1 (2020): 10. doi: 10.1128/jmbe.v21i1.2071 32313599 PMC7148151

[24] Haynes Chayla. "Dismantling the White Supremacy Embedded in Our Classrooms: White Faculty in Pursuit of More Equitable Educational Outcomes for Racially Minoritized Students." International Journal of Teaching and Learning in Higher Education 29, no. 1 (2017): 87–107.

[25] Marini Jessica P., Emily J. Shaw, and Linda Young. "Using Old and New SAT® Scores for Admission: A Closer Look at Concordant Scores in Predictive Models. Research Report 2016–17." College Board (2016).

[26] Wasserstein Ronald L., Schirm Allen L. & Lazar Nicole A. (2019) Moving to a World Beyond “p < 0.05”, The American Statistician, 73:sup1, 1–19, doi: 10.1080/00031305.2019.1583913

[27] Zwick Rebecca. "Is the SAT a “wealth test”? The link between educational achievement and socioeconomic status." Rethinking the SAT. Routledge, 2013. 225–238.

[28] Mattern K. D., Shaw E. J., and Williams F.E. Examining the Relationship between the SAT®, High School Measures of Academic Performance, and Socioeconomic Status: Turning Our Attention to the Unit of Analysis. Research Notes. (2008) RN-36. *College Board*.

[29] Zwick Rebecca, and Igor Himelfarb. "The effect of high school socioeconomic status on the predictive validity of SAT scores and high school grade‐point average." *Journal of Educational Measurement* 48.2 (2011): 101–121.

[30] Hurtado Sylvia, June C. Han, Victor B. Sáenz, Lorelle L. Espinosa, Nolan L. Cabrera, and Oscar S. Cerna. "Predicting transition and adjustment to college: Biomedical and behavioral science aspirants’ and minority students’ first year of college." Research in Higher Education 48, no. 7 (2007): 841–887.

[31] Ironside Pamela M. "“Covering content” and teaching thinking: Deconstructing the additive curriculum." *Journal of Nursing Education* 43.1 (2004): 5–12. 14748529 10.3928/01484834-20040101-02

[32] Bauerlein Mark. "Protecting College Students from Uncomfortable Ideas: On campus, feelings and fear triumph over thought." *Education Next* 19.2 (2019): 76–78.

[33] Blackburn James. "Understanding Paulo Freire: Reflections on the origins, concepts, and possible pitfalls of his educational approach." *Community Development Journal* 35.1 (2000): 3–15.

[34] Arnett Jeffrey Jensen. "Emerging adulthood: A theory of development from the late teens through the twenties." *American psychologist* 55.5 (2000): 469. 10842426

