# Supplementary material for: Inclusive and active pedagogies reduce academic outcome gaps and improve long-term performance
Source: PLoS One. 2022 Jun 15;17(6):e0268620. doi: 10.1371/journal.pone.0268620 (PMC9200326; doi:10.1371/journal.pone.0268620)
Supplement: S1 File — (DOCX) [file pone.0268620.s002.docx]

Supplemental Tables (S1)

Complex sample design (clustered by year and section) but with the removal of first-generation status (n = 3927, df = 19, pseudo-R^2^ = 0.377).

| Variable | OR | p-value |
| --- | --- | --- |
| Treatment | 1.727 | 0.03 |
| URM | 1.045 | 0.442 |
| NSATC | 0.994 | <.001 |
| HSGPA | 0.192 | <.001 |
| URM*treatment | --- | 0.25 |

Complex sample design (clustered by section only, n = 3077, df = 3, pseudo-R^2^ = 0.387).

| Variable | OR | p-value |
| --- | --- | --- |
| Treatment | 2.263 | <.001 |
| FG | 0.834 | 0.066 |
| URM | 1.217 | 0.583 |
| NSATC | 0.994 | 0.001 |
| HSGPA | 0.202 | 0.003 |
| URM*treatment | --- | 0.02 |
| FG*treatment | --- | 0.964 |

Complex sample design (clustered by section only) but with the removal of first-generation status (n = 4129, df = 3, pseudo-R^2^ = 0.380).

| Variable | OR | p-value |
| --- | --- | --- |
| Treatment | 1.744 | 0.006 |
| URM | 1.035 | 0.15 |
| NSATC | 0.994 | <.001 |
| HSGPA | 0.205 | <.001 |
| URM*treatment | --- | 0.13 |

Complex sample design (clustered by section only, n = 2887, df = 3, pseudo-R^2^ = 0.396).

| Variable | OR | p-value |
| --- | --- | --- |
| Treatment | 1.714 | 0.007 |
| FG | 0.789 | 0.043 |
| URM | 1.357 | 0.631 |
| NSATC | 0.994 | 0.002 |
| HSGPA | 0.182 | 0.006 |
| Year | --- | --- |
| URM*treatment | --- | 0.025 |
| FG*treatment | --- | 0.633 |
| Year*treatment | --- | 0.046 |

Complex sample design (clustered by section only) but with the removal of first-generation status (n = 3927, df = 3, pseudo-R^2^ = 0.382).

| Variable | OR | p-value |
| --- | --- | --- |
| Treatment | 1.242 | 0.009 |
| URM | 1.144 | 0.467 |
| NSATC | 0.994 | <.001 |
| HSGPA | 0.194 | 0.001 |
| Year | --- | 0.029 |
| URM*treatment | --- | 0.096 |
| Year*treatment | --- | 0.018 |
